# Adult-onset Still’s disease: evaluation of prognostic tools and validation of the systemic score by analysis of 100 cases from three centers

**DOI:** 10.1186/s12916-016-0738-8

**Published:** 2016-12-01

**Authors:** Piero Ruscitti, Paola Cipriani, Francesco Masedu, Daniela Iacono, Francesco Ciccia, Vasiliki Liakouli, Giuliana Guggino, Francesco Carubbi, Onorina Berardicurti, Paola Di Benedetto, Marco Valenti, Giovanni Triolo, Gabriele Valentini, Roberto Giacomelli

**Affiliations:** 1 Rheumatology Section, Department of Biotechnological and Applied Clinical Science, School of Medicine, University of L’Aquila, Delta 6 Building, Via dell’Ospedale, 67100 L’Aquila, Italy; 2 Medical Statistic Section, Department of Biotechnological and Applied Clinical Science, School of Medicine, University of L’Aquila, L’Aquila, Italy; 3 Rheumatology Section, Department of Clinical and Experimental Medicine, Second University of Naples, Naples, Italy; 4 Rheumatology Section, Department of Internal Medicine, University of Palermo, Palermo, Italy

**Keywords:** Adult-onset Still’s disease, Systemic score, Macrophage activation syndrome, Prognostic factor

## Abstract

**Background:**

Adult-onset Still’s disease (AOSD) is rare inflammatory disease of unknown etiology that usually affects young adults. The more common clinical manifestations are spiking fevers, arthritis, evanescent rash, elevated liver enzymes, lymphadenopathy, hepatosplenomegaly, and serositis. The multi-visceral involvement of the disease and the different complications, such as macrophage activation syndrome, may strongly decrease the life expectancy of AOSD patients.

**Methods:**

This study aimed to identify the positive and negative features correlated with the outcome of patients. A retrospective analysis of AOSD patients prospectively admitted to three rheumatologic centers was performed to identify the clinical features present at the time of diagnosis and to predict the possible outcome. Furthermore, we investigated the as yet to be validated prognostic value of the systemic score previously proposed.

**Results:**

One hundred consecutive AOSD patients were enrolled. The mean systemic score showed that the majority of patients had a multi-organ involvement. Sixteen patients showed different complications, mainly the macrophage activation syndrome. A strong increase of inflammatory markers was observed. All patients received steroids at different dosages, 55 patients in association with immunosuppressive drugs and 32 in association with biologic agents. Sixteen patients died during the follow-up. Regression analysis showed that the higher values of the systemic score and the presence of AOSD-related complications, assessed at the time of diagnosis, were significantly correlated with patient mortality. A prognostic impact of the systemic score of ≥ 7.0 was reported.

**Conclusions:**

Our study showed that a higher systemic score and the presence of AOSD-related complications at the time of diagnosis were significantly associated with mortality. Of note, a cut-off at 7.0 of the systemic score showed a strong prognostic impact in identifying patients at risk of AOSD-related death.

## Background

Adult-onset Still’s disease (AOSD) is a rare, systemic, inflammatory disorder of unknown etiology with an estimated incidence of 0.14–0.40 cases per 100,000 people and a prevalence of 1–34 cases per million people [1, 2]. It affects young adults, with a higher prevalence in women [2–4] and most commonly presents with high daily spiking fever, arthritis and evanescent rash. Other clinical features include sore throat, elevated liver enzymes, lymphadenopathy, hepatosplenomegaly, and serositis [5–7].

The diagnosis is often delayed because of the low specificity of most findings. However, the favorable effects of an early diagnosis on prognosis have been underlined [8]. Basically, three different clinical patterns of AOSD have been identified: (1) monocyclic pattern, characterized by a systemic single episode; (2) polycyclic pattern, characterized by multiple flares lasting for a 1 year or longer, alternating with remissions; and (3) chronic pattern, related to a persistently active disease with associated polyarthritis [9]. Usually, 30% of AOSD patients develop a monocyclic pattern, 30% a polycyclic pattern, and 40% a chronic pattern [2]. The monocyclic and polycyclic patterns have been considered as part of the systemic form of AOSD. On the contrary, the persistently active disease with associated chronic polyarthritis takes the articular form, suggesting that the underlying immunological imbalance might be different between these forms and could partially explain the reported differences in effectiveness of different therapeutic agents [10–13]. Moreover, AOSD patients may experience several severe complications associated with a decrease in life expectancy, such as macrophage activation syndrome (MAS), thrombotic thrombocytopenic purpura, respiratory distress syndrome, and diffuse alveolar hemorrhage [14–21].

In patients with AOSD, laboratory tests reflect the systemic inflammatory process and high levels of both erythrocyte sedimentation rate (ESR) and C-reactive protein (CRP). In addition, serum ferritin levels are much higher than those observed in other autoimmune, inflammatory, infectious, or neoplastic diseases, characterized by decreased glycosylated ferritin (<20%) [22, 23]. Depite the poor specificity, a 5-fold increase of serum ferritin levels are strongly suggestive of AOSD and, furthermore, it is generally considered a useful marker to assess the activity of the disease [24, 25].

The treatment of AOSD remains largely empirical, lacking controlled clinical trials [26]. Systemic corticosteroids are usually the first line therapy when systemic symptoms predominate, and often in combination with synthetic disease-modifying anti-rheumatic drugs (sDMARDs) such as methotrexate (MTX) [26]. In the last years, many biological agents, mainly interleukin (IL)-1 and IL-6 inhibitors, have been successfully used in refractory cases [10, 11].

At present, only few studies have focused on the prognostic factors of the disease [2, 4–6]. Moreover, most studies were single center studies based on a limited number of patients. To overcome these limitations, we planned a retrospective analysis of patients prospectively admitted to three different rheumatologic centers. Our study clearly identified the clinical features present at the time of diagnosis, predicting the possible different patient outcomes. Furthermore, we investigated the thus far non-validated prognostic value of the systemic score proposed by Pouchot et al. in 1991 [27].

## Methods

A total of 100 AOSD patients who fulfilled at least five (two major criteria and three minor criteria) of the Yamaguchi diagnostic criteria [28] and who were consecutively admitted to three rheumatologic centers from January 1, 2000, to December 31, 2015 were enrolled. The rheumatologic centers were selected by their expertise on management of AOSD and in the inception cohort studies. In this study, we could not use Fautrel’s criteria for AOSD diagnosis because none of the healthcare facilities associated with our university hospitals evaluates glycosylated ferritin levels [1, 23]. Although the specificity of Fautrel’s criteria is higher (98.5%), this datum is balanced by the higher sensitivity of the used criteria (Yamaguchi’s criteria sensitivity 96.2% vs. Fautrel’s criteria sensitivity 80.6%) [1, 23, 28].

The following clinical features at diagnosis were recorded: fever, typical rash, arthralgia or arthritis, myalgia, lymphadenopathy, sore throat, splenomegaly, hepatomegaly or abnormal liver function tests, abdominal pain, sore throat, weight loss, and gastrointestinal symptoms. Pleural effusion or pleuritis and lung parenchymal involvement were evaluated by a chest radiograph or CT scan, and pericarditis was evaluated by echocardiography. Table 1 shows the main demographic findings of our patients. The clinical workup before the AOSD diagnosis considered the exclusion of potential mimickers [1, 2]. We excluded infections by blood cultures and, in MAS patients, bone marrow cultures, serology, PCR analyses, chest X-rays, and abdominal echography. We evaluated the possible differential diagnosis with malignancies using chest X-rays, abdominal echography, and blood samples. Despite these exams, in the case of further suspicion, we added CT and/or PET/CT exams to the diagnostic workup. With regards to patients with possible hematologic cancers, we also performed bone marrow examination and lymph node biopsy. Autoimmune diseases were excluded by blood tests, antinuclear antibodies, anti-citrullinated peptide autoantibodies, rheumatoid factor, and anti-neutrophil cytoplasmic antibodies, and for the exclusion of systemic vasculitides we included tissue biopsy and arteriography in the workup. Finally, we evaluated a possible differential diagnosis with autoinflammatory diseases by the execution of gene analyses.

**Table 1:** Demographic and clinical features of the enrolled patients at the time of diagnosis

Clinical data	Patients
Women/men	66/34
Age, years ± SD	45.35 ± 16.23
Systemic score, mean ± SD	6.11 ± 2.02
Comorbidities, number (%)	33 (33)
Outcome	
Favorable outcome, number (%)	84 (84)
Monocyclic course	29 (29)
Polycyclic course	22 (22)
Chronic course	33 (33)
Unfavorable outcome, number (%)	16 (16)
AOSD-related death	16 (16)
Time of follow-up, years (mean ± SD)	3.53 ± 2.93
Median survival time (25%, 75%), years	2.5 (1.8–5)

Each patient was assessed for the systemic score proposed by Pouchot et al. [27] for AOSD. This score assigns 1 point to each of 12 manifestations: fever, typical rash, pleuritis, pneumonia, pericarditis, hepatomegaly or abnormal liver function tests, splenomegaly, lymphadenopathy, leukocytosis > 15,000/mm^3^, sore throat, myalgia, and abdominal pain (maximum score: 12 points). It has been used as a score for systemic disease [6, 27, 29], but its predictive value has never been investigated thus far.

In addition, at the time of diagnosis, each patient was characterized for the presence of AOSD-related complications such as MAS, thrombotic thrombocytopenic purpura, thrombotic microangiopathy, disseminated intravascular coagulopathy, respiratory distress syndrome, diffuse alveolar hemorrhage, pulmonary arterial hypertension, myocarditis, tamponade, constrictive pericarditis, endocarditis, shock, multiple organ failure, fulminant hepatitis, and amyloidosis, as suggested by the available literature [14]. MAS was defined following the diagnostic criteria proposed by the Histiocyte Society in 1991 and updated in 2004 and by Fardet L. et al. [30–33]. In AOSD-related MAS patients, the prognostic factors were assessed [34].

In our cohort, we investigated the presence of comorbidities at the time of diagnosis. Comorbidities were defined as coexisting medical conditions distinct from the principal diagnosis for which the patient was enrolled in this study [35].

Treatment regimens used in the course of disease were categorized into four groups, i.e., low/medium dose of steroids: ≤ 0.5 mg/kg/day of prednisone; high dose of steroids as > 0.5 mg/kg/day of prednisone; combination therapy with steroids plus sDMARD(s); and steroids plus biologics agent with or without sDMARD(s).

According to the disease course, at the last scheduled visit, patients were divided into four groups as described by Cush et al. [9]: the three classic clinical patterns (monocyclic, polycyclic, and chronic) and death, whichever the course. A monocyclic course was defined as a single episode for more than 2 months but less than 1 year followed by sustained remission through the entire follow-up period. A polycyclic course was characterized by recurrent systemic flares with remission between flares. A chronic course was defined as at least one episode of persistent symptoms lasting longer than 1 year. Expired patients were defined as those who were diagnosed with AOSD and died during follow-up. AOSD-related death was defined as death associated with AOSD or its complications during the follow-up. Moreover, remission was defined as the complete disappearance of systemic symptoms and normalization of laboratory evidence of disease activity for at least two consecutive months, regardless of therapy. Flare was characterized by systemic fares occurring after remission [9, 27]. In this study, we considered the need of any additional treatments and/or any increased dosages of drugs as a flare of the disease. The correlations between any disease feature and outcome were investigated.

The local ethics committee (Azienda Sanitaria Locale 1 Avezzano/Sulmona/L’Aquila, L’Aquila, Italy; protocol number 0139815/16) approved the procedure, which was performed according to the Good Clinical Practice guidelines and to the Declaration of Helsinki.

### Statistical analysis

An observational prospective design was set in order to provide a risk model profile for AOSD patients. Due to the relatively simple design and the expertise of the recruiting centers, we did not have missing data that could impair our analyses. The statistical analysis provided preliminary descriptive statistics both for supposed predictors and for the AOSD-dependent variable assumed as response. An exploratory χ^2^ analysis and exploratory logistic univariate analysis were carried out to clarify the possible associations and provide indications to select candidate predictors. The correlation between complications and the other selected clinical variables has been estimated using a point biserial correlation with the corresponding *P* values. An ordered logistic risk model, adjusted by sex and age, was performed to provide odds ratio estimations for the independent variables previously selected. The assumed latent linear behavior of the response variable underwent a Brandt test (*P* < 0.05). The overall model fitting was estimated using a likelihood ratio test (LR = 91.55, *P* < 0.0001), using the empty one as a reference model. According to the inferential analysis performed in the ordinal logistic regression, the variable AOSD was dichotomized: dead versus alive patients, patients with monocyclic pattern versus other, patients with polycyclic pattern versus other, patients with chronic pattern versus other. This strategy allowed the study, exploiting logistic regressions, of the significant predictors detected in the general ordinal logistic model. Due to the low number of AOSD-related complications, at the time of diagnosis, we did not perform any statistical analysis for single complications. Thus, we decided to aggregate these covariates into a single dichotomous variable.

The analysis suggested a reasonable clinical application of the systemic score as predictor of the disease condition upshot. The analysis dealt with this issue categorizing the AOSD outcome variable into a dichotomous variable, which divided patients into dead or alive. A statistical association between AOSD death and the systemic score was preliminarily assessed (χ^2^ (df = 10) = 23.32; *P* = 0.01). Then, a receiver operating characteristic (ROC) validation analysis was carried out, obtaining an overall performance of the test in terms of the area under the ROC curve (AUC = 0.80 ± 0.06). The test sensitivity and specificity was estimated according to the Youden criteria, obtaining a systemic score cut-off of 7.0. The last step tested the consistency of this cut-off in discriminating the survival of those having systemic score above and below 7.0 performing a Kaplan–Meyer estimate of the corresponding survivor functions and a log-rank test to assess the statistically significant difference between the estimated survival functions (log-rank χ^2^ = 11.94, *P* = 0.0005). Statistical analysis was performed using the statistical software STATA, version 14.

## Results

### Clinical features

**Fig. 1:**
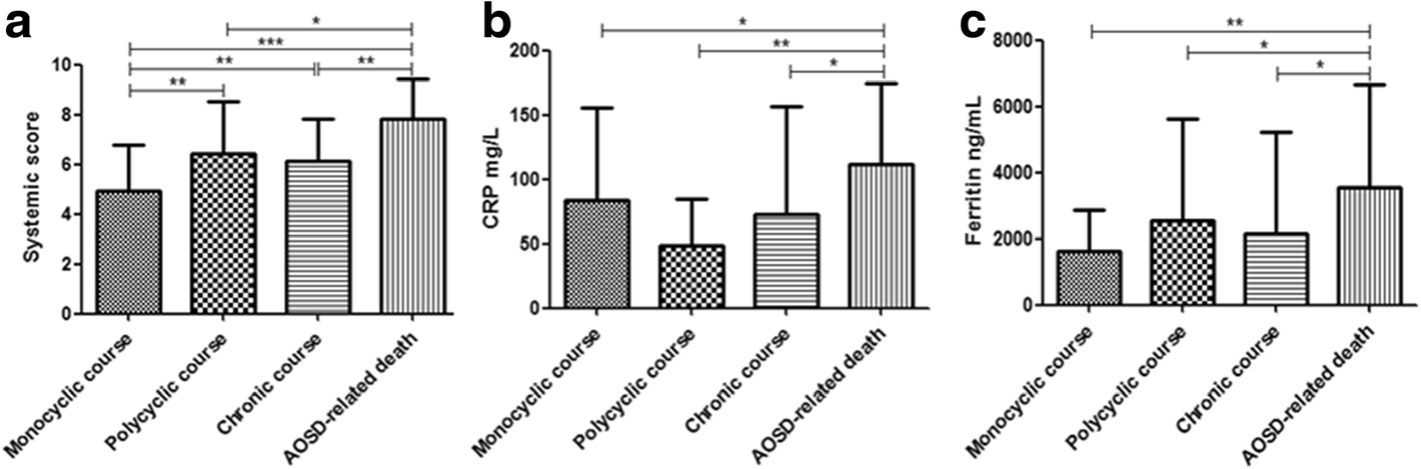
Clinical and laboratory differences among the groups with different clinical outcomes. **a** Panel shows the values of the systemic score, at the time of diagnosis, among the different groups according the clinical outcome, the highest values are observed in the AOSD-related death group. **b** and **c** Panels show the serum levels of C-reactive protein and ferritin, at the time of diagnosis; these levels are statistically higher in AOSD-related death group when compared with the other groups. Values are expressed as mean ± SD (*P < 0.05; **P < 0.001; ***P < 0.0001).

One hundred consecutive AOSD patients (66 men, 34 women), whose age at diagnosis was 45.35 ± 16.23 (mean ± SD), were enrolled (Table 1). Table 2 compares the clinical and laboratory features of patients assessed at admission. Briefly, all patients experienced fever, 86 showed joint involvement, 78 presented with a skin rash, 62 showed hepatic involvement, and 57 experienced myalgia. In addition, 79 and 57 patients presented with splenomegaly and lymph node enlargement, respectively. A low percentage of patients experienced serositis, pericarditis, pleurisy, and weight loss. The mean systemic score resulted in 6.11 ± 2.02 (mean ± SD, range 2–12, median 6), indicating that the majority of patients had multi-organ involvement. In Fig. 1, we report the statistically significant differences among the groups with different clinical outcomes.

**Table 2:** Demographic and clinical features of the evaluated patients stratified according the different outcomes

	All patients	Monocyclic course	Polycyclic course	Chronic course	AOSD-related death
Number of patients	100	29	22	33	16
Female/Male	66/34	20/9	12/10	12/11	12/4
Age (years), mean ± SD	45.35 ± 16.23	49.31 ± 12.47	43.81 ± 15.13	40.24 ± 15.86	50.81 ± 19.97
*Clinical features*					
Fever, n (%)	100 (100)	29 (100)	22 (100)	33 (100)	16 (100)
Weight loss, n (%)	5 (5)	1 (3.44)	2 (9.10)	0	2 (12.5)
Rash, n (%)	78 (78)	24 (82.75)	14 (48.27)	28 (84.84)	12 (75)
Joints involvement, n (%)	86 (86)	27 (93.10)	21 (95.45)	26 (78.79)	12 (75)
Sore throat, n (%)	64 (64)	10 (34.48)	13 (59.10)	25 (75.76)	16 (100)
Myalgia, n (%)	57 (57)	12 (41.37)	15 (68.19)	16 (48.49)	14 (87.5)
Lymphadenopathy, n (%)	57 (57)	11 (37.93)	16 (72.72)	15 (45.45)	15 (93.75)
Splenomegaly, n (%)	79 (79)	22 (75.86)	20 (90.91)	23 (69.70)	14 (87.5)
Hepatic involvement, n (%)	62 (62)	18 (62.07)	16 (72.72)	18 (54.54)	10 (62.5)
Pleurisy, n (%)	14 (14)	1 (3.44)	5 (22.72)	4 (12.12)	4 (25)
Lung involvement, n (%)	13(13)	2 (6.89)	3 (13.63)	1 (3.03)	7 (43.75)
Pericarditis, n (%)	15 (15)	1 (3.44)	3 (13.63)	6 (18.18)	5 (31.25)
Abdominal pain, n (%)	18 (18)	2 (6.89)	4 (18.19)	7 (21.21)	5 (31.25)
Systemic score, mean ± SD	6.11 ± 2.02	4.93 ± 1.79	6.41 ± 2.05	6.12 ± 1.66	7.81 ± 1.54
Comorbidities, n (%)	33 (33)	6 (20.68)	8 (36.36)	9 (27.28)	10 (62.5)
*Laboratory markers*					
Leukocytosis > 15,000/mm^3^, n (%)	36 (36)	4 (13.79)	7 (31.82)	19 (57.57)	6 (37.5)
Serum ferritin (ng/mL), mean ± SD	2560.07 ± 3726.64	1634.82 ± 1198.92	2541.04 ± 3028.16	2886.09 ± 5438.65	3560.94 ± 3004.93
ESR (mm/hour), mean ± SD	67.28 ± 26.65	68.93 ± 23.94	61.81 ± 26.84	65.18 ± 26.77	74.93 ± 28.29
CRP (mg/L), mean ± SD	78.35 ± 72.76	83.83 ± 70.38	48.34 ± 35.64	72.16 ± 83.47	111.37 ± 61.76
*Complications*					
MAS	13 (13)	0	3 (13.63)	0	10 (62.5)
Kidney failure	2 (2)	0	0	0	2 (12.5)
Myocarditis	1 (1)	1 (3.44)	0	0	0
*Treatments*					
Low dosage of steroid monotherapy, n (%)	4 (4)	0	0	4 (12.12)	0
High dosage steroid monotherapy, n (%)	39 (39)	24 (79.31)	2 (9.1)	7 (21.21)	6 (93.75)
High dosage steroid pulse therapy (500–1000 mg), n (%)	37 (37)	10 (34.48)	9 (40.91)	5 (15.15)	13 (81.25)
sDMARD(s)	55 (55)	5 (17.24)	18 (81.82)	22 (66.67)	10 (62.5)
Combination therapy, steroids + sDMARD(s), n (%)	25 (25)	5 (17.24)	8 (36.36)	10 (30.3)	2 (12.5)
Combination therapy, steroids + biologics agent ± sDMARD(s), n (%)	32 (32)	0	12 (54.55)	12 (36.37)	8 (50)

*ESR* erythrocyte sedimentation rate, *CRP* C-reactive protein, *MAS* macrophage activation, *sDMARDs* synthetic disease-modifying anti-rheumatic drugs

Sixteen patients experienced different complications at the time of diagnosis. Specifically, 13 patients were affected by AOSD-related MAS at the time of diagnosis. Bone marrow analysis showed the phagocytosis of haematopoietic cells by activated macrophages in all of these patients. The clinical features of these patients are listed in Table 3. Two patients experienced kidney failure requiring dialysis in whom an interstitial nephritis occurred, as assessed by kidney biopsies. Finally, one patient experienced myocarditis as an AOSD-related complication. Our analysis showed that higher values of the systemic score were significantly associated with the presence of AOSD-related complications at the time of diagnosis. On the contrary, no correlation was found between ESR, CRP, and/or serum ferritin levels and the presence of AOSD-related complications at the time of diagnosis (Table 4).

**Table 3:** Demographic and clinical features of the enrolled patients with macrophage activation syndrome (MAS)

Clinical features	
Number of patients	13
Women/men	7/6
Age (years), mean ± SD	52.02 ± 19.01
*Triggering factor*	
AOSD flare, number (%)	13 (100)
Lymphoma, number (%)	0
Infectious disease, number (%)	0
*Laboratory features*	
WBC (10^3^/mL), mean ± SD	3.28 ± 1.36
RBC (10^3^/mL), mean ± SD	3.31 ± 0.69
HB (gr/dL), mean ± SD	9.19 ± 2.05
PLT (10^3^/mL), mean ± SD	55.52 ± 46.54
Serum Ferritin (ng/mL), mean ± SD	4362.15 ± 7569.70
ESR (mm/hour), mean ± SD	71.23 ± 30.19
CRP (mg/L), mean ± SD	74.76 ± 46.92
Triglycerides (mg/dL), mean ± SD	183.12 ± 69.33
ASAT (IU/L), mean ± SD	74.59 ± 41.89
ALAT (IU/L), mean ± SD	129.98 ± 91.21
*Treatments*	
High dosage steroid pulses, number (%)	13 (100)
Immunosuppressive drugs, number (%)	7 (53.84)
Cyclosporine A, number (%)	5 (38.46)
Methotrexate, number (%)	2 (15.38)
Etoposide	0
Biologic drugs	2 (15.38)
Deaths, number (%)	10 (76.92)
Number of relapses in MAS-survivors patients, mean ± SD	2.66 ± 1.69

*AOSD* adult-onset Still’s disease, *IU* international unit, *WBC* white blood cell count, *RBC* red blood cells, *HB* hemoglobin, *PLT* platelet count, *ESR* erythrocyte sedimentation rate, *CRP* C-reactive protein, *ASAT* aspartate aminotransferase, *ALAT* alanine aminotransferase

**Table 4:** Correlation between AOSD-related complications and selected clinical variables

Clinical variables	Coefficient, *P* value
Systemic score	0.40, < 0.001*
Serum ferritin	0.13, 0.20
Erythrocyte sedimentation rate	0.06, 0.56
C-reactive protein	0.18, 0.07

*Statistically significant

In this cohort, a strong increase of inflammatory markers (mean ± SD) was observed: serum ferritin levels were 2560.07 ± 3726.64 ng/mL, ESR was 67.28 ± 26.65 mm/hour, and CRP was 78.35 ± 72.76.

With regards to comorbidities at baseline, 33 patients presented at least one comorbidity. In fact, patients were affected by systemic arterial hypertension (*n* = 21), dyslipidemia (*n* = 10), thyroidopathies (*n* = 10), type 2 diabetes (*n* = 8), osteoporosis (*n* = 5), hepatic steatosis (*n* = 5), atrial fibrillation (*n* = 2), heart failure (*n* = 2), and cardiac valvular disease (*n* = 2). In addition, seven patients were affected by chronic kidney disease with mildly reduced kidney function (Glomerular Filtration Rate = 60–89 mL/min/1.73 m^2^) [36]. Chronic kidney disease was associated with type 2 diabetes (*n* = 3), systemic arterial hypertension (*n* = 3), and polycystic kidney disease (*n* =1).

The monocyclic pattern, polycyclic, and chronic patterns were present in 29, 22, and 33 patients, respectively. In patients affected by polycyclic pattern, a mean of 2.89 ± 0.86 flares occurring after remission and requiring additional therapy were observed during follow-up. Sixteen patients died during the follow-up period. Specifically, 10 patients died of uncontrollable MAS, two of severe kidney failure requiring dialysis, two of multiple organ failure, and two of severe infection related to the immunosuppressive therapy.

### Treatments

All patients received steroids at different dosages, the mean dosage of prednisone equivalent was 321.81 ± 394.52 (mean ± SD) mg. Table 2 shows the percentage of patients treated by pulse steroid therapy. The design of our observational study did not establish any prior therapeutic strategy or the tapering regimen of steroids. We followed the general rule of commencing steroid tapering once the maximum desired therapeutic benefit has been obtained, when inadequate therapeutic benefit has been achieved following an adequate therapeutic strategy, or when side effects, such as type 2 diabetes or hypertension, become serious or uncontrollable with medication [37]. Four patients were treated with low/medium dose of steroid monotherapy, whereas 39 patients were treated by high dose steroid monotherapy. Fifty-five patients were treated with a combination therapy, including sDMARD(s) and steroids: patients received MTX (*n* = 43), cyclosporine A (*n* = 8), hydroxychloroquine (*n* = 4), cyclophosphamide (*n* = 2), a combination therapy with MTX and cyclosporine A (*n* = 5), and MTX and hydroxychloroquine (*n* = 2). Thirty-two patients were treated with a combination therapy including biologic agents and/or sDMARD and/or steroids: patients received TNF inhibitors (*n* = 18), tocilizumab (*n* = 8), and anakinra (*n* = 6). Among patients treated with biologic agents, 29 were treated with a combination therapy with biologic agent and sDMARD and steroids, and three were treated with a combination therapy biologic agent and steroids. Twenty-one out of 32 patients treated with biologic agents received high dose steroids. The above results are summarized in Table 5.

**Table 5:** Treatments of enrolled Adult-onset Still’s disease patients at the time of diagnosis

Treatment	Patients
Steroids, number (%)	100 (100)
Low dose of steroids monotherapy, number (%)	4 (4)
High dose of steroids monotherapy, number (%)	39 (39)
Steroid pulse therapy, number (%)	37 (37)
sDMARD, number (%)	55 (55)
Methotrexate	43 (43)
Cyclosporine A	8 (8)
Hydroxychloroquine	4 (4)
Cyclophosphamide	2 (2)
Methotrexate and cyclosporine A	5 (5)
Methotrexate and hydroxychloroquine	2 (2)
Biologic agents, number (%)	32 (32)
Infliximab	10 (10)
Tocilizumab	8 (8)
Etanercept	7 (7)
Anakinra	6 (6)
Certolizumab pegol	1 (1)
Combination therapy, steroids + sDMARD(s), number (%)	25 (25)
Combination therapy, steroids + biologics agent ± sDMARD(s), number (%)	32 (32)

*sDMARDs* synthetic disease-modifying anti-rheumatic drugs

### Regression analyses among clinical features at the time of diagnosis and outcomes

An ordinal regression analysis was performed to estimate whether sex, systemic score, the presence of AOSD-related complications, the presence of comorbidities, serum ferritin levels, and inflammatory markers, at the time of diagnosis, were associated with outcome of our patients (Table 6). Due to the low number of AOSD-related complications at the time of diagnosis we decided to aggregate these covariates into a single dichotomous variable in order to minimize the possible confounding effect of the low number of patients on the statistical analyses. In addition, the aggregation of these covariates might improve the generalization of the results and thus their clinical usability.

**Table 6:** Ordinal regression analysis between clinical features at the time of diagnosis and outcomes

	Odds ratio	Standard error	*P*	95% confidence interval
Gender	1.40	0.66	0.48	0.55–3.54
Systemic score	1.42	0.17	0.011*	1.12–1.81
AOSD-related complication	51.57	40.61	<0.0001*	11.02–241.36
Comorbidities	3.89	1.94	0.021*	1.46–10.35
Serum Ferritin	1.00	0.00	0.57	1.00–1.00
Erythrocyte sedimentation rate	0.99	0.01	0.54	0.98–1.01
C-reactive protein	0.99	0.00	0.07	0.99–1.00
Time of observation	0.92	0.08	0.37	0.78–1.10

*AOSD* Adult-onset Still’s disease

*Statistically significant

Our results showed that the higher values of the systemic score, the presence of AOSD-related complication, and the presence of comorbidities were associated with the outcome. The results suggest that higher values of the systemic score or the presence of comorbidities at the time of diagnosis were predictive of a more severe outcome than the monocyclic form (Table 7). Furthermore, the higher values of the systemic score or the presence of AOSD-related complications at the time of diagnosis were significantly associated with mortality in our cohort, our study showed that AOSD-related complications are the main clinical features negatively influencing the survival of AOSD patients.

**Table 7:** Regression analyses among clinical features at the time of diagnosis and different outcomes

	Odds ratio	Standard error	*P*	95% confidence interval
*Monocyclic pattern*				
Systemic score	0.60	0.13	0.019*	0.39–0.92
AOSD-related complication	0.32	0.38	0.34	0.03–3.34
Comorbidities	0.13	0.11	0.017*	0.02–0.69
*Polycyclic pattern*				
Systemic score	1.13	0.14	0.31	0.89–1.46
AOSD-related complication	1			
Comorbidities	2.82	1.52	0.049*	0.98–8.16
*Chronic pattern*				
Systemic score	1.01	0.15	0.90	0.76–1.36
AOSD-related complication	1.84	1.35	0.40	0.43–7.81
Comorbidities	1.12	0.66	0.84	0.35–3.61
*AOSD-related death*				
Systemic score	1.49	0.30	0.04*	1.00–2.23
AOSD-related complication	33.52	34.63	0.001*	4.22–253.92
Comorbidities	1.38	1.35	0.34	0.20–9.36

*AOSD* Adult-onset Still’s disease

*Statistically significant

### Validation of the systemic score

In our cohort, we observed that higher levels of the systemic score were predictive of a more severe outcome than the monocyclic form. On this basis, we supposed that a reasonable clinical application of the systemic score, as predictor of the disease condition, might be proposed. Thus, a specific statistical analysis was performed in order to validate this score.

**Fig. 2:**
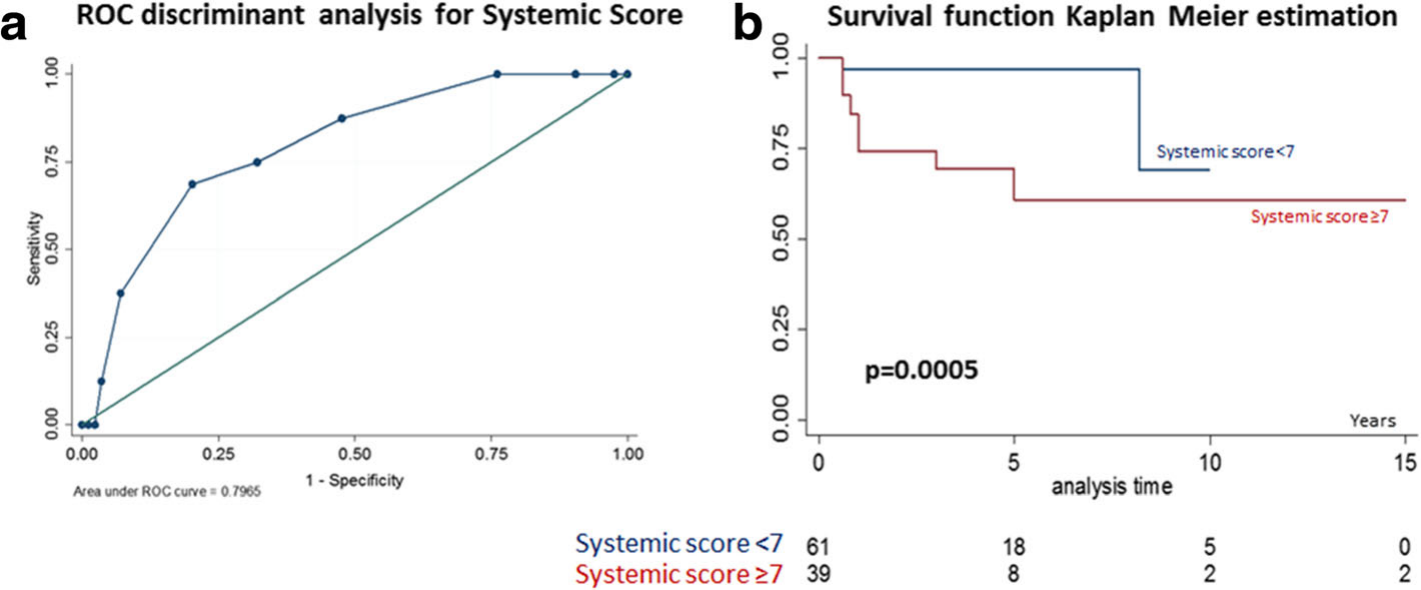
Kaplan–Meier estimates of survival according to systemic score. **a** The panel shows the ROC analysis validation for the systemic score. It was performed with an overall performance of the test in terms of the area under the ROC curve (AUC = 0.80 ± 0.06, 95% CI, 0.68–0.91). The sensitivity and specificity were estimated according the Youden criteria, obtaining a systemic score cut-off of 7.0 (sensitivity: 75.00%; specificity: 67.86%). **b** The panel shows the prognostic impact of systemic score ≥ 7.0. The Kaplan–Meier analysis showed a significant association between a systemic score ≥ 7.0 and mortality (*P* = 0.0005)

A statistical association between AOSD-related death and the systemic score was preliminarily assessed (χ^2^ (df = 10) = 23.32; *P* = 0.01). Then, a ROC analysis validation was performed, leading to an overall performance of the test in terms of the area under the ROC curve (AUC = 0.80 ± 0.06, 95% CI, 0.68–0.91). The test sensitivity and specificity was estimated according to the Youden criteria, obtaining a systemic score cut-off of 7.0 (systemic score ≥ 7.0; sensitivity: 75.00%; specificity: 67.86%). The last step was to test the consistency of this cut-off in discriminating the survival between patients having the systemic score upper and lower of 7.0, performing a Kaplan–Meyer estimate of the corresponding survivor functions and a log-rank test to assess the statistically significant difference between the survival functions estimated (log-rank χ^2^ = 11.94, *P* = 0.0005). Finally, Kaplan–Meier analysis showed a significant association between the systemic score ≥ 7.0 and mortality (*P* = 0.0005). The prognostic impact of systemic score ≥ 7.0 is shown in Fig. 2.

## Discussion

AOSD is a rare systemic inflammatory disorder, characterized by a higher mortality rate. Despite increasing progress in unraveling the pathogenesis of the disease, evidence-based data are still lacking and, consequently, the disease course is unpredictable at onset and the therapeutic approach is still largely anecdotal [2, 10, 11, 14]. To our knowledge, our study is the first observational study in a large cohort of AOSD patients to investigate the prognostic factors present at the time of diagnosis that may be associated with different outcomes and the prognostic impact of systemic score ≥ 7.0.

As far as the clinical picture is concerned, all our patients experienced fever, frequently associated with both joint and skin involvement, mirroring data previously published [5, 6, 8, 27, 38–41]. In fact, it has been reported that fever occurs in 60–100% of AOSD patients, typically with the highest temperatures occurring in the evening. Fever usually precedes the onset of other manifestations [2, 3]. Joint involvement is reported in 70–100% of these patients, mainly in wrists, knees, and ankles. At the onset of disease, arthritis may be mild and transient, sometimes evolving into a chronic destructive symmetrical polyarthritis with carpal ankylosis [5, 6, 8]. Further, 60–80% of AOSD patients experience a macular or maculopapular evanescent salmon-pink skin rash associated with the fever spiking [38–41]; some patients, those with a usually more severe outcome, may experience this skin involvement for many weeks [7, 42]. Similar to previous studies [22, 24], a strong increase of inflammatory markers as well as of serum ferritin levels were observed herein [43, 44], confirming the systemic nature of the disease. Although it has been proposed that AOSD might be included within the so called “Hyperferritinemic Syndrome”, a common umbrella gathering different diseases in which the increased circulating ferritin levels might not only reflect an acute phase response but be directly involved in inflammation [25], our study did not find any correlation between the levels of this molecule and the patient outcomes.

With regards to the complications, it must be noted that 13 patients simultaneously fulfilled the diagnostic criteria for AOSD and MAS, a life-threatening complication of several diseases, including AOSD, at the time of diagnosis [15–17]. Our results confirmed previous papers in which up to 16% of AOSD patients experienced this complication [5, 6, 14]. Interestingly, the clinical features of these MAS patients mirrored those reported in available literature [15, 16, 34]. Our patients showed peripheral cytopenias associated with high levels of both inflammatory markers and serum ferritin. All these patients were treated with a steroid pulse therapy [43, 45, 46]. Of note, it has been proposed that AOSD and MAS may be part of the same disease spectrum, in which AOSD should be considered the milder form [14], and the occurrence of MAS might be misdiagnosed due to the immunosuppressive treatments used to control an AOSD flare [14, 15]. AOSD may be associated with life-threatening manifestations, such as myocarditis, adult respiratory distress syndrome, and pulmonary hypertension [14]. Although the prognosis of AOSD is generally favorable, death may occur in some patients due to these complications, and early recognition and management of these conditions is associated with decreased mortality in these patients [14]. In addition, our analysis showed that the higher values of the systemic score, a score evaluating the multi-visceral involvement, were associated with the presence of AOSD-related complications.

A favorable outcome was showed in the majority of patients: 29% out of the enrolled patients experienced the monocyclic pattern and maintained remission during follow-up, 55% developed a chronic form with a polycyclic (22%) or chronic pattern (33%).

In our study, we focused on the prognostic tools at the time of diagnosis and this validated severity score may be applied to all the AOSD patients, identifying, at the time of diagnosis, those patients with the likelihood of a more severe outcome. In fact, our study confirms that a cut-off at 7.0 of the systemic score at the time of diagnosis identifies those patients at higher risk of AOSD-related death. Our results suggest that multi-organ involvement at the time of diagnosis is predictive of a more severe outcome and increased mortality.

Furthermore, our study suggests that not only multi-organ involvement but also the presence of comorbidities at the time of diagnosis may be predictive of the more severe outcomes. In fact, it is well-known from epidemiological studies that patients with comorbid illnesses, independently from the primary disease, may be at higher risk of complications or death, less able to tolerate specific procedures, and less responsive to therapy, when compared to patients with the same primary disease but without these conditions [29, 47].

In our cohort, we observed 16 AOSD-related deaths; uncontrollable MAS was the most frequent complication associated with death. Our study suggests that higher values of the systemic score and the presence of AOSD-related complications at the time of diagnosis were significantly associated with mortality, negatively influencing survival. The higher mortality rate observed herein does not confirm results from other European series in which a lower mortality rate was reported [27, 40], but mirrors recently published data observed in Asiatic populations reporting a similar mortality rate as well as the main causes of death [48, 49].

When we analyzed the AOSD treatments at the time of diagnosis, we observed that the majority of patients were treated with combination therapy including steroids and MTX, the latter showing both steroid-sparing effect and effectiveness [38–40, 50, 51]. In fact, it is well-known that both steroids and MTX present strong anti-inflammatory and immunosuppressive effects [52–54]. Recently, multiple lines of evidence reported the efficacy of biologic agents in controlling AOSD clinical symptoms in steroids- and sDMARD-refractory AOSD [55–57]. In our cohort, according with the suggestions of Jamilloux et al. [58], patients resistant to “traditional” therapies were treated with TNF-inhibitors or with IL-1 or IL-6 antagonists. We did not analyze any possible association between the use of biological treatments, as well as the other therapeutic schemes, and the different outcomes. Our study was not specifically designed to analyze the effect of different drugs on the outcome. Furthermore, this may result in a “confounding by indication” bias, i.e., patients treated with biologics were the most severe ones [59, 60]. Thus, lacking international guidelines for AOSD treatment, it is probable that clinicians decided to prescribe a more intensive treatment to the patients that, in their opinion, were affected by a more aggressive disease [59, 60]. Confounding by indication is a potential limitation in the follow-up of observational studies to evaluate the possible effects of different therapeutic strategies, thus strongly limiting the interpretation of the results [59–61]. In addition, it must be noted that this kind of study generally may display some further limitations; in fact, although we performed a retrospective analysis of patients prospectively admitted to three different rheumatologic centers, some confounding factors, such as misclassification or information biases, might be present in any retrospective series.

## Conclusions

In conclusion, our study showed that a higher systemic score and/or the presence of AOSD-related complications at the time of diagnosis were significantly associated with a poor, unfavorable outcome. Of note, this works is the first demonstration that a systemic score cut-off of 7.0 has a strong prognostic impact, identifying patients at risk of AOSD-related death. The results of our study, identifying the prognostic factors which may be detectable at the time of diagnosis and validating a severity score for the disease, may be helpful for the clinical management of AOSD patients in the future.

## Abbreviations

AOSD: adult-onset Still’s disease
CRP: C-reactive protein
ESR: erythrocyte sedimentation rate
IL: interleukin
MAS: macrophage activation syndrome
MTX: methotrexate
sDMARDs: synthetic disease-modifying anti-rheumatic drugs

## References

[1] Mahroum N, Mahagna H, Amital H (2014). Diagnosis and classification of adult Still’s disease. J Autoimmun.

[2] Gerfaud-Valentin M, Jamilloux Y, Iwaz J, Sève P (2014). Adult-onset Still’s disease. Autoimmun Rev..

[3] Cagatay Y, Gul A, Cagatay A, Kamali S, Karadeniz A, Inanc M (2009). Adult-onset Still’s disease. Int J Clin Pract..

[4] Sampalis JS, Esdaile JM, Medsger TA, Partridge AJ, Yeadon C, Senécal JL (1995). A controlled study of the long-term prognosis of adult Still’s disease. Am J Med..

[5] Kong X-D, Xu D, Zhang W, Zhao Y, Zeng X, Zhang F (2010). Clinical features and prognosis in adult-onset Still’s disease: a study of 104 cases. Clin Rheumatol..

[6] Kim YJ, Koo BS, Kim Y-G, Lee C-K, Yoo B (2013). Clinical features and prognosis in 82 patients with adult-onset Still’s disease. Clin Exp Rheumatol..

[7] Cozzi A, Papagrigoraki A, Biasi D, Colato C, Girolomoni G (2016). Cutaneous manifestations of adult-onset Still’s disease: a case report and review of literature. Clin Rheumatol..

[8] Kalyoncu U, Solmaz D, Emmungil H, Yazici A, Kasifoglu T, Kimyon G (2016). Response rate of initial conventional treatments, disease course, and related factors of patients with adult-onset Still’s disease: Data from a large multicenter cohort. J Autoimmun..

[9] Cush JJ, Medsger TA, Christy WC, Herbert DC, Cooperstein LA (1987). Adult-onset Still’s disease. Clinical course and outcome. Arthritis Rheum.

[10] Maria AT, Le Quellec A, Jorgensen C, Touitou I, Rivière S, Guilpain P (2014). Adult onset Still’s disease (AOSD) in the era of biologic therapies: dichotomous view for cytokine and clinical expressions. Autoimmun Rev..

[11] Kontzias A, Efthimiou P (2008). Adult-onset Still’s disease: pathogenesis, clinical manifestations and therapeutic advances. Drugs..

[12] Chen DY, Lan JL, Lin FJ, Hsieh TY (2004). Proinflammatory cytokine profiles in sera and pathological tissues of patients with active untreated adult onset Still’s disease. J Rheumatol..

[13] Canna SW (2014). Editorial: Interferon-γ: friend or foe in systemic juvenile idiopathic arthritis and adult-onset Still’s disease?. Arthritis Rheumatol..

[14] Efthimiou P, Kadavath S, Mehta B (2014). Life-threatening complications of adult-onset Still’s disease. Clin Rheumatol..

[15] Arlet JB, Le TH, Marinho A, Amoura Z, Wechsler B, Papo T (2006). Reactive haemophagocytic syndrome in adult-onset Still’s disease: a report of six patients and a review of the literature. Ann Rheum Dis..

[16] Ramos-Casals M, Brito-Zerón P, López-Guillermo A, Khamashta MA, Bosch X (2014). Adult haemophagocytic syndrome. Lancet..

[17] Kumakura S, Murakawa Y (2014). Clinical characteristics and treatment outcomes of autoimmune-associated hemophagocytic syndrome in adults. Arthritis Rheumatol..

[18] Perez MG, Rodwig FR (2003). Chronic relapsing thrombotic thrombocytopenic purpura in adult onset Still’s disease. South Med J..

[19] Gopal M, Cohn CD, McEntire MR, Alperin JB (2009). Thrombotic thrombocytopenic purpura and adult onset Still’s disease. AmJ Med Sci..

[20] Zheng XL, Kaufman RM, Goodnough LT, Sadler JE (2004). Effect of plasma exchange on plasma ADAMTS13 metalloprotease activity, inhibitor level, and clinical outcome in patients with idiopathic and nonidiopathic thrombotic thrombocytopenic purpura. Blood..

[21] Cheema GS, Quismorio FP (1999). Pulmonary involvement in adult-onset Still’s disease. Curr Opin Pulm Med..

[22] Pay S, Türkçapar N, Kalyoncu M, Simşek I, Beyan E, Ertenli I (2006). A multicenter study of patients with adult-onset Still’s disease compared with systemic juvenile idiopathic arthritis. Clin Rheumatol..

[23] Fautrel B, Le Moël G, Saint-Marcoux B, Taupin P, Vignes S, Rozenberg S (2001). Diagnostic value of ferritin and glycosylated ferritin in adult onset Still’s disease. J Rheumatol..

[24] Wang W, Knovich MA, Coffman LG, Torti FM, Torti SV (2010). Serum ferritin: past, present and future. Biochim Biophys Acta..

[25] Rosário C, Zandman-Goddard G, Meyron-Holtz EG, D’Cruz DP, Shoenfeld Y (2013). The hyperferritinemic syndrome: macrophage activation syndrome, Still’s disease, septic shock and catastrophic antiphospholipid syndrome. BMC Med..

[26] Jamilloux Y, Gerfaud-Valentin M, Martinon F, Belot A, Henry T, Sève P (2015). Pathogenesis of adult-onset Still’s disease: new insights from the juvenile counterpart. Immunol Res..

[27] Pouchot J, Sampalis JS, Beaudet F, Carette S, Décary F, Salusinsky-Sternbach M (1991). Adult Still’s disease: manifestations, disease course, and outcome in 62 patients. Medicine (Baltimore).

[28] Yamaguchi M, Ohta A, Tsunematsu T, Kasukawa R, Mizushima Y, Kashiwagi H (1992). Preliminary criteria for classification of adult Still’s disease. J Rheumatol..

[29] Colafrancesco S, Priori R, Alessandri C, Perricone C, Pendolino M, Picarelli G (2012). IL-18 serum level in adult onset Still’s disease: a marker of disease activity. Int J Inflam..

[30] Filipovich AH. Hemophagocytic lymphohistiocytosis (HLH) and related disorders. Hematology Am Soc Hematol Educ Program. 2009:127–31. 10.1182/asheducation-2009.1.127 20008190

[31] Henter JI, Elinder G, Ost A (1991). Diagnostic guidelines for hemophagocytic lymphohistiocytosis. The FHL Study Group of the Histiocyte Society. Semin Oncol.

[32] Henter JI, Horne A, Aricó M, Egeler RM, Filipovich AH, Imashuku S, et al. HLH-2004: Diagnostic and therapeutic guidelines for hemophagocytic lymphohistiocytosis. Pediatr Blood Cancer. 2007;48:124–31. 10.1002/pbc.21039 16937360

[33] Fardet L, Galicier L, Lambotte O, Marzac C, Aumont C, Chahwan D (2014). Development and validation of the HScore, a score for the diagnosis of reactive hemophagocytic syndrome. Arthritis Rheumatol..

[34] Arca M, Fardet L, Galicier L, Rivière S, Marzac C, Aumont C (2015). Prognostic factors of early death in a cohort of 162 adult haemophagocytic syndrome: impact of triggering disease and early treatment with etoposide. Br J Haematol..

[35] Klabunde CN, Potosky AL, Legler JM, Warren JL (2000). Development of a comorbidity index using physician claims data. J Clin Epidemiol..

[36] Levey AS, de Jong PE, Coresh J, El Nahas M, Astor BC, Matsushita K (2011). The definition, classification, and prognosis of chronic kidney disease: a KDIGO Controversies Conference report. Kidney Int..

[37] Volkmann ER, Rezai S, Tarp S, Woodworth TG, Furst DE (2013). We still don’t know how to taper glucocorticoids in rheumatoid arthritis, and we can do better. J Rheumatol.

[38] Fautrel B, Zing E, Golmard JL, Le Moel G, Bissery A, Rioux C (2002). Proposal for a new set of classification criteria for adult-onset Still disease. Medicine (Baltimore).

[39] Colina M, Zucchini W, Ciancio G, Orzincolo C, Trotta F, Govoni M (2011). The evolution of adult-onset Still disease: an observational and comparative study in a cohort of 76 Italian patients. Semin Arthritis Rheum..

[40] Gerfaud-Valentin M, Maucort-Boulch D, Hot A, Iwaz J, Ninet J, Durieu I (2014). Adult onset Still disease: manifestations, treatments, outcome, and prognostic factors in 57 patients. Medicine (Baltimore).

[41] Sfriso P, Priori R, Valesini G, Rossi S, Montecucco CM, D’Ascanio A (2016). Adult-onset Still’s disease: an Italian multicentre retrospective observational study of manifestations and treatments in 245 patients. Clin Rheumatol..

[42] Ruscitti P, Cipriani P, Ciccia F, Di Benedetto P, Liakouli V, Berardicurti O (2016). H-ferritin and CD68(+)/H-ferritin(+) monocytes/macrophages are increased in the skin of adult-onset Still’s disease patients and correlate with the multi-visceral involvement of the disease. Clin Exp Immunol..

[43] Ruscitti P, Cipriani P, Di Benedetto P, Ciccia F, Liakouli V, Carubbi F (2015). Increased level of H-ferritin and its imbalance with L-ferritin, in bone marrow and liver of patients with adult onset Still’s disease, developing macrophage activation syndrome, correlate with the severity of the disease. Autoimmun Rev..

[44] Ruscitti P, Ciccia F, Cipriani P, Guggino G, Di Benedetto P, Rizzo A (2016). The CD68(+)/H-ferritin(+) cells colonize the lymph nodes of the patients with adult onset Still’s disease and are associated with increased extracellular level of H-ferritin in the same tissue: correlation with disease severity and implication for pathogenesis. Clin Exp Immunol..

[45] Ruscitti P, Cipriani P, Ciccia F, Masedu F, Liakouli V, Carubbi F (2016). Prognostic factors of macrophage activation syndrome, at the time of diagnosis, in adult patients affected by autoimmune disease: Analysis of 41 cases collected in 2 rheumatologic centers. Autoimmun Rev.

[46] Batu ED, Erden A, Seyhoğlu E, Kilic L, Büyükasık Y, Karadag O, et al. Assessment of the HScore for reactive haemophagocytic syndrome in patients with rheumatic diseases. Scand J Rheumatol. 2016;30:1–5. doi:10.3109/03009742.2016.1167951. 10.3109/03009742.2016.1167951 27359073

[47] Iezzoni LI, Foley SM, Daley J, Hughes J, Fisher ES, Heeren T (1992). Comorbidities, complications, and coding bias: does the number of diagnosis codes matter in predicting in-hospital mortality?. JAMA..

[48] Kim H-A, Sung J-M, Suh C-H (2012). Therapeutic responses and prognosis in adult-onset Still’s disease. Rheumatol Int..

[49] Zeng T, Zou Y-Q, Wu M-F, Yang C-D (2009). Clinical features and prognosis of adult-onset Still’s disease: 61 cases from China. J Rheumatol..

[50] Franchini S, Dagna L, Salvo F, Aiello P, Baldissera E, Sabbadini MG (2010). Efficacy of traditional and biologic agents in different clinical phenotypes of adult-onset Still’s disease. Arthritis Rheum..

[51] Fautrel B, Borget C, Rozenberg S, Meyer O, Le Loët X, Masson C (1999). Corticosteroid sparing effect of low dose methotrexate treatment in adult Still’s disease. J Rheumatol..

[52] Spies CM, Bijlsma JW, Burmester GR, Buttgereit F (2010). Pharmacology of glucocorticoids in rheumatoid arthritis. Curr Opin Pharmacol..

[53] Cipriani P, Ruscitti P, Carubbi F, Liakouli V, Giacomelli R (2014). Methotrexate: an old new drug in autoimmune disease. Expert Rev Clin Immunol..

[54] Gerards AH, de Lathouder S, de Groot ER, Dijkmans BA, Aarden LA (2003). Inhibition of cytokine production by methotrexate. Studies in healthy volunteers and patients with rheumatoid arthritis. Rheumatology.

[55] Giampietro C, Fautrel B (2012). Anti-interleukin-1 agents in adult onset Still’s disease. Int J Inflam..

[56] Ortiz-Sanjuán F, Blanco R, Calvo-Rio V, Narvaez J, Rubio Romero E, Olivé A (2014). Efficacy of tocilizumab in conventional treatment-refractory adult-onset Still’s disease: multicenter retrospective open-label study of thirty-four patients. Arthritis Rheumatol..

[57] Cipriani P, Ruscitti P, Carubbi F, Pantano I, Liakouli V, Berardicurti O (2014). Tocilizumab for the treatment of adult-onset Still’s disease: results from a case series. Clin Rheumatol..

[58] Jamilloux Y, Gerfaud-Valentin M, Henry T, Sève P (2014). Treatment of adult-onset Still’s disease: a review. Ther Clin Risk Manag..

[59] Landewé R, van der Heijde D (2002). Follow up studies in rheumatoid arthritis. Ann Rheum Dis..

[60] Salas M, Hofman A, Stricker BH (1999). Confounding by indication: an example of variation in the use of epidemiologic terminology. Am J Epidemiol..

[61] Signorello LB, McLaughlin JK, Lipworth L, Friis S, Sørensen HT, Blot WJ (2002). Confounding by indication in epidemiologic studies of commonly used analgesics. Am J Ther..

